# What every intensivist should know about acute respiratory distress syndrome and diffuse alveolar damage

**DOI:** 10.5935/0103-507X.20170044

**Published:** 2017

**Authors:** Fernando Rios, Teresa Iscar, Pablo Cardinal-Fernández

**Affiliations:** 1 Department of Critical Care, Hospital Nacional Alejandro Posadas - Buenos Aires, Argentina.; 2 Department of Pathology, Hospital Universitario HM Puerta del Sur - Madrid, Spain.; 3 Department of Emergency, Hospital Universitario HM Sanchinarro - Madrid, Spain.

**Keywords:** Acute respiratory distress syndrome, Diffuse alveolar damage, Pharmacological treatment, Surrogate biomarkers

## Abstract

Acute respiratory distress syndrome is a challenging entity for the intensivist. The pathological hallmark of the acute phase is diffuse alveolar damage, which is present in approximately half of living patients with acute respiratory distress syndrome. It is clear that respiratory support for acute respiratory distress syndrome has gradually been improving over recent decades. However, it is also evident that these procedures are beneficial, as they reduce lung injury and keep the patient alive. This could be interpreted as a time-gaining strategy until the trigger or causal or risk factor improves, the inflammatory storm decreases and the lung heals. However, all except two pharmacological treatments (neuromuscular blockers and steroids) were unable to improve the acute respiratory distress syndrome outcome. The hypothesis that pharmacological negative results may be explained by the histological heterogeneity of acute respiratory distress syndrome has been supported by the recent demonstration that acute respiratory distress syndrome with diffuse alveolar damage constitutes a specific clinical-pathological entity. Given that diffuse alveolar damage is a pathological diagnosis and that open lung biopsy (the most common technique to obtain lung tissue) has several side effects, it is necessary to develop surrogate biomarkers for diffuse alveolar damage. The aim of this narrative review is to address the following three topics related to acute respiratory distress syndrome: (a) the relationship between acute respiratory distress syndrome and diffuse alveolar damage, (b) how diffuse alveolar damage could be surrogated in the clinical setting and (c) how enrichment in diffuse alveolar damage may improve the results of pharmacological clinical trials tried out on patients with acute respiratory distress syndrome.

## Abstract (pt)

A síndrome do desconforto respiratório agudo é um desafio para o intensivista. A característica principal desta doença aguda é o dano alveolar difuso, presente em cerca de metade dos pacientes com a síndrome. É claro que o suporte respiratório à síndrome do desconforto respiratório agudo tem melhorado gradualmente nas últimas décadas. É também evidente que todos estes procedimentos são benéficos, já que reduzem a lesão pulmonar e mantêm o paciente vivo. Isto deve ser interpretado como uma estratégia de ganho de tempo, até que o fator desencadeante ou de risco causal melhore, assim como a tempestade inflamatória diminua e o pulmão se cure. Por outro lado, todos - exceto dois tratamentos farmacológicos (bloqueadores neuromusculares e esteroides) - são incapazes de melhorar o desfecho da síndrome do desconforto respiratório agudo. A hipótese de que os resultados farmacológicos negativos podem ser explicados pela heterogeneidade histológica da síndrome do desconforto respiratório agudo tem sido apoiada pelas recentes demonstrações de que a síndrome com dano alveolar difuso tem característica clínico-patológica específica. O dano alveolar difuso é um diagnóstico patológico, e a biópsia pulmonar a céu aberto (a técnica mais comum para obtenção de tecido pulmonar) tem efeitos colaterais graves, sendo necessário que se desenvolvam biomarcadores substitutos para o dano alveolar difuso. O objetivo desta revisão é discutir três tópicos relacionados à síndrome do desconforto respiratório agudo: o relacionamento entre a síndrome do desconforto respiratório agudo e o dano alveolar difuso; como o dano alveolar difuso pode ser representado no quadro clínico; e como o enriquecimento pode melhorar os resultados de estudos clínicos farmacológicos realizados com pacientes com a síndrome e com dano alveolar difuso.

## INTRODUCTION

Nearly half a century after its first description,^(1)^ acute respiratory distress syndrome (ARDS) continues to be one of the most relevant life-threatening entities in critically ill patients. Despite the great scientific and economic efforts humanity has made to improve ARDS outcome, a recent global survey demonstrated that ARDS has a prevalence of 0.42 cases per intensive care unit (ICU) bed and a mortality rate of 40%.^(2)^ In addition, the clinical management of ARDS has improved dramatically, but this improvement is based on techniques (e.g., low tidal volume or low pressure plateau) where the most likely main effect is to avoid lung injury associated with mechanical ventilation. Except for early paralyzation and likely steroids, all pharmacological treatments tried on patients with ARDS were unable to demonstrate a relevant effect.^(3,4)^

Diffuse alveolar damage (DAD) is considered the histological hallmark for the acute phase of ARDS.^(5)^ It has been well known for many years that DAD is present in only half of autopsies from patients with ARDS.^(6,7)^ However, the recent demonstration that the same proportion occurs in living patients,^(8)^ as well as the effect that DAD exerts over ARDS outcome, shine a new light on this entity.^(9-11)^

The aim of this narrative review is to address three topics about ARDS. First, we address the relationship between ARDS and DAD. Second, we analyze how DAD could be surrogated in the clinical setting. Finally, we address how enrichment in DAD may improve the results of clinical trials tried out on ARDS patients.

### What is the relationship between acute respiratory distress syndrome and diffuse alveolar damage?

According to the Berlin definition,^(5)^ ARDS is a clinical construct composed by (i) the presence of at least one risk factor associated to (ii) acute hypoxemia not fully explained by cardiac failure or fluid overload and (iii) bilateral infiltration on radiology. On the other hand, the American Thoracic Society/European Respiratory Society International Multidisciplinary Consensus Classification of the Idiopathic Interstitial Pneumonias^(12,13)^ defined two histological, indistinguishable patterns: the acute interstitial pneumonia (AIP) and the DAD. The former term, AIP, is reserved for cases of unknown causes, and the latter term, DAD, is for patients with ARDS. In other words, both terms exhibit the same pathological pattern but differ in the clinical context in which they are diagnosed. The aforementioned consensus defined DAD (or AIP) by the presence of key histological features (diffuse distribution, uniform temporal appearance, alveolar septal thickening due to organizing fibrosis, usually diffuse airspace organization may be patchy or diffuse, hyaline membranes) and pertinent negative findings (lack of granulomas, necrosis, or abscesses, lack of infectious agents, no viral inclusions and negative results with special stains for organisms, lack of prominent eosinophils and neutrophils and negative cultures).

**Figure 1:**
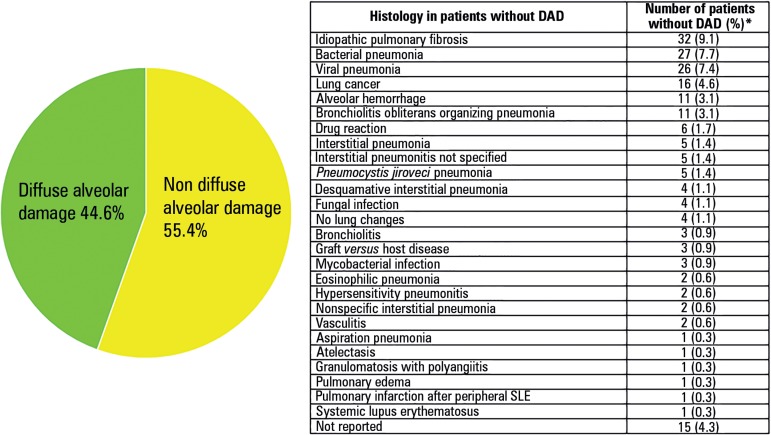
Histological finding in open lung biopsies performed in patients with acute respiratory distress syndrome.^(8)^ DAD - diffuse alveolar damage; SLE - systemic lupus erythematosus. * The percentage was calculated using the whole acute respiratory distress syndrome cohort (n = 350).

Although it is not unanimously accepted,^(14,15)^ the Berlin definition considered DAD as the hallmark for the acute phase of ARDS.^(5)^ This discrepancy may be explained by (i) the fact that a high proportion of the knowledge related to the ARDS pathology has been derived from autopsy studies, (ii) the effect of DAD on the ARDS outcome was unknown and (iii) what occurred in patients with mild ARDS was not described.^(3,15,16)^ In addition, the complexity of diagnosing DAD in patients with ARDS (see below) creates a great challenge for its study.^(14)^ Despite all of these difficulties, recently, several advances have been reported in understanding the relationship between ARDS and DAD. First, it was demonstrated that approximately half of living patients with ARDS present DAD in the pathological analysis of lung tissue obtained with an open lung biopsy.^(8)^ The other half showed one among a number of heterogeneous diseases (Figure 1), some of them with a specific treatment in the case of being diagnosed (e.g., pneumonia, pulmonary embolism or carcinomatous lymphangitis). Second, the effect of DAD on ARDS outcome was demonstrated in post-mortem and living patients. Lorente et al.^(11)^ analyzed 150 autopsies from patients with ARDS and found that the presence of DAD was associated with a lower age, lower ratio of partial oxygen pressure and inspiratory fraction (Pa02/Fi02), and lower respiratory dynamic compliance, as well as a higher punctuation in the sequential organ failure score (SOFA) scale.

Of paramount importance was the fact that the cause of death was associated with the histological finding (in patients without DAD, refractory shock was the main cause of death in 55% and refractory hypoxemia in 5%; in contrast, in patients with DAD, refractory shock was the main cause of death in 29% and refractory hypoxemia in 25%). Similar differences were found between patients with ARDS and DAD *versus* ARDS with histological pneumonia. Cardinal-Fernández et al.^(8)^ analyzed 350 living patients with ARDS and open lung biopsy. They found that, although no differences were observed in the severity of the patients with and without DAD (Pa0_2_/Fi0_2_ and SOFA punctuation were similar on the day that the ARDS diagnosis and open lung biopsy were performed), mortality in patients with DAD was almost double than in patients without DAD (OR 1.81; IC95% 1.14 - 2.86). Kao et al.^(10)^ found that DAD is an independent risk factor for hospital mortality in living patients with ARDS (OR 3.55; IC95% 1.38 - 9.12). Finally, given that pneumonia (viral and bacterial) is the second most frequent histological finding in patients with ARDS (Figure 1), it has been postulated that DAD and histological pneumonia could be considered together, with the aim to increase the correlation between clinical and pathological findings.^(17)^

Although inconclusive, several facts argue against this proposal: (a) from a pathological point of view, DAD and pneumonia constitute two different entities that may exist independently from each other, (b) the microbiological rate of isolation differs in both entities,^(18)^ (c) the clinical evolution and the cause of death are different^(11)^ and (d) the mortality rate is also different.^(10)^ However, all of these differences do not exclude the possibility that some physiopathological pathways may be present in both entities and might explain why some pharmacological treatments may improve both conditions (see below).

### How can diffuse alveolar damage be diagnosed?

Based on the previously described evidence, it appears necessary to recognize the subgroup of patients with ARDS and DAD with the aim to define a clinical-pathological entity,^(3,11,16,19)^ to increase the correlation between clinical and histological findings and to develop personalized pharmacological treatments (see below).^(20-22)^ Currently, the only model to estimate the probability of presenting DAD in patients with ARDS has been developed and validated in autopsies, and its accuracy is just moderate (area under receive operative curve 0.74, IC95% 0.65 -0.82).^(11)^ Likewise, the most frequent procedure to diagnose the DAD is performing an open lung biopsy, which is a risky procedure reserved for centers with demonstrated experience. Open lung biopsy is only recommended in two scenarios: (a) when there is high suspicion of curable etiology, less invasive procedures (e.g., bronchoalveolar lavage, blood samples and CT scan) are inconclusive and the risk of empirical therapy is too high and/or (b) when it is considered necessary to identify the fibro-proliferative phase (towards the end of the first week of evolution) to prescribe steroids.^(15,23,24)^

The problem of diagnosing the gold standard is common to numerous diseases (e.g., myocardial infarction, neurodegenerative diseases and osteoporosis) and can be resolved using surrogate biomarkers. A biomarker is a characteristic that is objectively measured and evaluated as an indicator of normal biological processes, pathogenic processes or biological responses to a therapeutic intervention."^(25)^ A surrogate endpoint is "a biomarker that is intended to substitute for a clinical endpoint. A surrogate endpoint is expected to predict clinical benefit (or harm) based on epidemiologic, therapeutic, pathophysiologic, or other scientific evidence."^(25)^

The most common types of biomarkers are based on measuring clinical parameters or molecules. Imaging techniques have also been successfully used as surrogate biomarkers. In recent years, the combination of structural (e.g., computer tomography or nuclear magnetic resonance) with functional (e.g., positron emission tomography) imaging techniques has determined the appearance of a new kind of biomarker called functional imaging, which allows for the understanding of how physiological (or physiopathological) processes occur in a specific structure of the body.

A surrogate biomarker for DAD should have particular characteristics such as: (a) high accuracy for the diagnosis of DAD as well as ruling out any other diseases that may mimic the ARDS (this statement determines that the discovery and validation of a surrogate biomarker for DAD has to be performed using pathological findings); (b) high precision (the result can not vary if the same sample is analyzed several times using the same technique and the same laboratory conditions); (c) reflect the stage of the DAD evolution; (d) correlate the amount of parenchyma with DAD and (e) with the response of a specific treatment for DAD.

Finally, each kind of biomarker presents specific requirements. For example, if it is a molecule, it should be (a) present in minimally invasive samples (e.g., blood, urine or bronchio-alveolar lavage); (b) simple (e.g., a unique molecule with different levels of cut-off); (c) measurable with laboratory equipment available in average hospitals; (d) able to allow results to be obtained in a brief period of time; and (e) easily interpreted by physicians at the bedside. In addition, if it is a causal factor for DAD, it is more relevant because it could also be considered a therapeutic target. If the biomarker is an imaging technique, it should (a) be able to be performed with minimal displacement of the patient; (b) in a short period of time; (c) allow for the maintenance of all treatment and monitoring and (d) avoid the use of contrast that could harm the patient.

At this moment, N-terminal-peptide type III procollagen (*NT*-*PCP*-*III*) appears to be the most plausible surrogate biomarker for the fibro-proliferative phase of patients with ARDS. Forel et al.^(26)^ conducted an elegant study which included 32 consecutive patients presenting non-resolving, moderate or severe ARDS and open lung biopsy. In the study, they assessed the NT-PCP-III in serum and bronchioalveolar lavage as a surrogate biomarker of the fibro-proliferative phase in patients with ARDS. They found that the NT-PCP-III, measured 3 days (median) before the open lung biopsy, was higher in patients with ARDS with fibro-proliferation than in ARDS without fibro-proliferation (area under ROC was 0.90 [95%CI 0.80 - 1.00] for bronchioalveolar lavage and 0.75 [95%CI 0.57 - 0.92] for serum).

### Why have almost all pharmacological treatments tried on acute respiratory distress syndrome failed?

A drug is usually defined as any chemical substance that affects the functioning of living things and organisms (e.g., bacteria, fungi, or viruses). Likewise, a drug target is "a molecular structure (chemically definable by at least a molecular mass) that will undergo a specific interaction with chemicals that we call drugs because they are administered to treat or diagnose a disease. The interaction has a connection with the clinical effect(s)."^(27)^

On the other hand, clinical trials are a type of experiment designed to answer a specific question related to biomedical or behavioral intervention, including new treatments, protocols or medical devices. Currently, under the term "ARDS" in the international database *Clinical Trials,* 58 studies (drugs 41, cell therapy 7 and biological therapy 10) appeared, including 8376 patients (Tables 1 and 2).^(28)^

**Table 1:** Studies based on pharmacologic treatment attempted in patients with acute respiratory distress syndrome and registered in a clinical trial database^(28)^

Type intervention	NCT Number	Drug	Title of the study	Sponsor or collaborators	Patients enrolled
Drug	NCT01504867	Acid acetilsalicilic	LIPS-A: Lung Injury Prevention Study with Aspirin	Ognjen Gajic/Beth Israel Deaconess Medical Center/Montefiore Medical Center/Vanderbilt University/Mayo Clinic	400
Drug	NCT01659307	Acid acetilsalicilic	The Effect of Aspirin on Reducing Inflammation in Human in Vivo Model of Acute Lung Injury	Belfast Health and Social Care Trust/The Intensive Care Society United Kingdom/Northern Ireland Clinical Trials Unit/Queen's University, Belfast	33
Drug	NCT00112164	Activated protein C	Activated Protein C to Treat Acute Lung Injuries	University of California, San Francisco/National Heart, Lung, and Blood Institute (NHLBI)	90
Drug	NCT02106975	Ascorbic acid	Vitamin C Infusion for Treatment in Sepsis Induced Acute Lung Injury	Virginia Commonwealth University/National Heart, Lung, and Blood Institute (NHLBI)	170
Drug	NCT01434121	Ascorbic acid	Ascorbic Acid (Vitamin C) Infusion in Human Sepsis	Virginia Commonwealth University	24
Drug	NCT01050699	Dexmedetomidine	Sleep Intervention During Acute Lung Injury	University of Arizona/National Heart, Lung, and Blood Institute (NHLBI)	90
Drug	NCT00351533	Fish oil	A Phase II Randomized Trial of Fish Oil in Patients with Acute Lung Injury (ALI)	University of Washington/National Heart, Lung, and Blood Institute (NHLBI)/American Thoracic Society|Acute Respiratory Distress Syndrome Foundation/American Society for Parenteral and Enteral Nutrition	90
Drug	NCT01335932	Gancliclovir/Valganciclovir	Study of Ganciclovir/Valganciclovir for Prevention of Cytomegalovirus Reactivation in Acute Injury of the Lung and Respiratory Failure	Fred Hutchinson Cancer Research Center/National Heart, Lung, and Blood Institute (NHLBI)/Genentech, Inc.	160
Drug	NCT01713309	Heparin binding protein	Heparin Binding Protein in Patients with Acute Respiratory Failure Treated with GCSF (Filgrastim)	Helsinki University Central Hospital/The Swedish Research Council	59
Drug	NCT02425579	Inhaled carbon monoxide	Safety Study of Inhaled Carbon Monoxide to Treat Acute Respiratory Distress Syndrome (ARDS)	Weill Medical College of Cornell University/Brigham and Women's Hospital/Massachusetts General Hospital/Duke University	48
Drug	NCT00605696	Insulin	Evaluating the Effectiveness of Early Insulin Therapy in People at Risk for Developing Acute Lung Injury/Acute Respiratory Distress Syndrome	National Heart, Lung, and Blood Institute (NHLBI)	90
Drug	NCT01096771	Intravenous lipids	The Effect of Intravenous Lipids on Lung Function in Acute Respiratory Distress Syndrome (ARDS)	Methodist Research Institute, Indianapolis	14
Drug	NCT01938079	Ketamine	Pharmacokinetic Alterations During ECMO	Columbia University	20
Drug	NCT00159510	Methylene blue & nitric oxide	Studies of Acute Lung Injury (ALI) and Acute Respiratory Distress Syndrome	Northern State Medical University/Helse Nord	28
Drug	NCT00655928	N-acetylcysteine	Modulation of Lung Injury Complicating Lung Resection	Imperial College London/Royal College of Physicians/Royal Brompton & Harefield NHS Foundation Trust	47
Drug	NCT01573715	Neuromuscular blocking agents	Effects of Neuromuscular Blocking Agents (NMBA) on the Alteration of Transpulmonary Pressures at the Early Phase of Acute Respiratory Distress Syndrome (ARDS)	Assistance Publique Hopitaux De Marseille	40
Drug	NCT00299650	Neuromuscular blocking agents	Systematic Early Use of Neuromuscular Blocking Agents in ARDS Patients	Assistance Publique Hopitaux de Marseille/GlaxoSmithKline	340
Drug	NCT02509078	Neuromuscular blocking agents	Reevaluation of Systemic Early Neuromuscular Blockade	Massachusetts General Hospital/National Heart, Lung, and Blood Institute (NHLBI)	1408
Drug	NCT00036062	Neutrophil elastase inhibitor	A Phase II Study to Determine the Efficacy and Safety of Sivelestat in Subjects with Acute Lung Injury	Eli Lilly and Company	600
Drug	NCT00219375	Neutrophil elastase inhibitor	Study of Sivelestat Sodium Hydrate in Acute Lung Injury (ALI) Associated with Systemic Inflammatory Response Syndrome (SIRS) in Japan	Ono Pharmaceutical Co. Ltd	649
Drug	NCT01391481	Perfluorocarbon inhaled	Perfluorocarbon (PFC) Inhalation Treatment of Acute Lung Injury/Acute Respiratory Distress Syndrome	Chinese PLA General Hospital/The Second Artillery General Hospital/The 306 Hospital of People's Liberation Army/First Hospitals affiliated to the China PLA General Hospital/General Hospital of Chinese Armed Police Forces/Beijing Shijitan Hospital/Air Force	200
Drug	NCT02370095	Prostaciclin analogue	Treprostinil Sodium Inhalation for Patients at High Risk for ARDS	---	NR
Drug	NCT01274481	Prostaciclin analogue	Iloprost Effects on Gas Exchange and Pulmonary Mechanics	University of Oklahoma/Actelion	20
Drug	NCT00455767	Protein inhibitor of human neutrophil elastase	Safety and Efficacy Study of Depelestat in Acute Respiratory Distress Syndrome (ARDS) Patients	Debiopharm International SA	84
Drug	NCT01597635	Recombinant human angiotensin converting enzyme type 2	The Safety, Tolerability, PK and PD of GSK2586881 in Patients with Acute Lung Injury	GlaxoSmithKline	43
Drug	NCT00996840	Selective inhibitor of p38 alpha (MAPK)	SB-681323 IV for Subjects at Risk of Acute Lung Injury or ARDS	GlaxoSmithKline	90
Drug	NCT02166853	Sevofluorane	Effects of SEvoflurane on Gas Exchange and Inflammation in Patients with ARDS (SEGA Study)	University Hospital, Clermont-Ferrand	50
Drug	NCT01619280	Sodium nitroprrusside	Safety Study of Nebulized Sodium Nitroprusside in Adult Acute Lung Injury	Mount Sinai Hospital, Canada	30
Drug	NCT00979121	Statins	Statins for Acutely Injured Lungs from Sepsis	National Heart, Lung, and Blood Institute (NHLBI)	745
Drug	NCT00562835	Steroids	Steroids in Patients with Early ARDS	Catholic University of the Sacred Heart	400
Drug	NCT00773058	Steroids	Effect of Treatment with Stress-Doses Glucocorticoid in Patients with Acute Respiratory Distress Syndrome (ARDS)	Southeast University, China/Nanjing Medical University	100
Drug	NCT01284452	Steroids	Efficacy of Hydrocortisone in Treatment of Severe Sepsis/Septic Shock Patients with Acute Lung Injury/Acute Respiratory Distress Syndrome (ARDS)	Mahidol University	197
Drug	NCT00290602	Steroids	Early Low Dose Steroid Therapy of Acute Respiratory Distress Syndrome	National Cancer Center, Korea	40
Drug	NCT01783821	Steroids	LIPS-B: Lung Injury Prevention Study with Budesonide and Beta	Mayo Clinic/Stanford University/Beth Israel Deaconess Medical Center/University of Arizona/National Center for Research Resources (NCRR)	61
Drug	NCT02819453	Steroids	Corticosteroid Mediates Acute Respiratory Distress Syndrome	Shanghai Pulmonary Hospital, Shanghai, China	20
Drug	NCT00127985	Steroids	6-Methyl-Prednisolone for Multiple Organ Dysfunction Syndrome	Hospital Universitario Principe de Asturias|Pfizer	240
Drug	NCT00742482	Surfactant	Efficacy and Safety of 3 Doses of HL10 Given at Fixed Time Intervals Compared to Standard Therapy	LEO Pharma	418
Drug	NCT01462279	Thiamine	Effect of Thiamine on Oxygen Utilization (VO2) in Critical Illness	Beth Israel Deaconess Medical Center/American Medical Association	20
Drug	NCT02895191	Urinary trypsin inhibitor	The Safety and Dose Response Relationship of Ulinastatin for Acute Respiratory Distress Syndrome (ARDS)	Techpool Bio-Pharma Co., Ltd./The First Affiliated Hospital of Guangzhou Medical University	60
Drug	NCT00004494	Vasoactive intestinal peptide	Phase I Study of Vasoactive Intestinal Peptide in Patients with Acute Respiratory Distress Syndrome and Sepsis	Stony Brook University/State University of New York/FDA Office of Orphan Products Development	18
Drug	NCT02468531	Xenon anesthesia	The Clinic Trial on Protection of Xenon Anaesthesia Against Perioperative Acute Lung Injury for Standford an Acute Aortic Dissection	Beijing Anzhen Hospital	80

Studies with one (NCT01814956 [lipid emulsions]) or not registered patients (NCT00030121 [recombinant human atrial natriuretic polypeptide], NCT00431379 [tissue plasminogen activator], NCT01713595 [inhaled saline], NCT02113735 [adrenocorticotropic hormone analogue] and NCT01195428 [simvastatin]) were not included in the table. NR - not reported.

**Table 2:** Studies based on cell and biological therapy conducted in patients with acute respiratory distress syndrome and registered in a clinical trial database^(28)^

Type of intervention	NCT number	Intervention	Title of the study	Sponsor or collaborators	Patients enrolled
Cell therapy	NCT02804945	Mesenchymal stem cells	Mesenchymal Stem Cells (MSCs) for Treatment of Acute Respiratory Distress Syndrome (ARD) in Stem Cell Transplant Patients	M.D. Anderson Cancer Center	50
Cell therapy	NCT01775774	Mesenchymal stem cells	Human Mesenchymal Stem Cells For Acute Respiratory Distress Syndrome	Michael A. Matthay/National Heart, Lung, and Blood Institute (NHLBI)/Massachusetts General Hospital/Stanford University/University of Pittsburgh/University of Minnesota - Clinical and Translational Science Institute/University of California, San Francisco	69
Cell therapy	NCT02097641	Mesenchymal stem cells	Human Mesenchymal Stem Cells for Acute Respiratory Distress Syndrome (START)	Michael A. Matthay/National Heart, Lung, and Blood Institute (NHLBI)/Massachusetts General Hospital/Stanford University/University of Pittsburgh/University of Minnesota - Clinical and Translational Science Institute/Ohio State University/University of Cal	60
Cell therapy	NCT02215811	Mesenchymal stem cells	Treatment of Severe Acute Respiratory Distress Syndrome with Allogeneic Bone Marrow-derived Mesenchymal Stromal Cells	Karolinska University Hospital/Karolinska Institutet	10
Cell therapy	NCT02444455	Mesenchymal stem cells	Human Umbilical-Cord-Derived Mesenchymal Stem Cell Therapy in Acute Lung Injury	Affiliated Hospital to Academy of Military Medical Sciences/Ivy Institute of Stem Cells Co. Ltd	20
Cell therapy	NCT02112500	Mesenchymal stem cells	Mesenchymal Stem Cell in Patients with Acute Severe Respiratory Failure	Asan Medical Center	10
Cell therapy	NCT02611609	Stem cells derived from bone marrow	A Phase 1/2 Study to Assess MultiStemÂ^®^ Therapy in Acute Respiratory Distress Syndrome	Athersys, Inc/Athersys Limited/Cell Therapy Catapult	36
Biological therapy	NCT01902082	Adipose-derived mesenchymal stem cells	Adipose-derived Mesenchymal Stem Cells in Acute Respiratory Distress Syndrome	Shaoxing Second Hospital	20
Biological therapy	NCT01438853	Anti-TF antibody	Effects of TNX-832 (Sunol cH36) in Subjects with Acute Lung Injury/Acute Respiratory Distress Syndrome	Altor Bioscience Corporation/Genentech, Inc./Tanox	18
Biological therapy	NCT00879606	Anti-tissue factor antibody	Anti-TF Antibody (ALT-836) to Treat Septic Patients with Acute Lung Injury or Acute Respiratory Distress Syndrome	Altor Bioscience Corporation/National Heart, Lung, and Blood Institute (NHLBI)	150
Biological therapy	NCT00233207	Chimeric CD14 antibody	IC14 Antibodies to Treat Individuals with Acute Lung Injury	National Heart, Lung, and Blood Institute (NHLBI)	13
Biological therapy	NCT00201409	Granulocyte macrophage colony-stimulating factor	A Randomized Trial of GM-CSF in Patients with ALI/ARDS	University of Michigan|National Heart, Lung, and Blood Institute (NHLBI)|Emory University/University of Colorado, Denver	132
Biological therapy	NCT02595060	Granulocyte macrophage colony-stimulating factor	Granulocyte Macrophage-Colony Stimulating Factor (GM-CSF) Inhalation to Improve Host Defense and Pulmonary Barrier Restoration	Savara Inc.	45
Biological therapy	NCT02095444	Human menstrual blood cells	Using Human Menstrual Blood Cells to Treat Acute Lung Injury Caused by H7N9 Bird Flu Virus Infection	S-Evans Biosciences Co.,Ltd./First Affiliated Hospital of Zhejiang University	20
Biological therapy	NCT02622724	Interferon beta-1a	Efficacy and Safety of FP-1201-lyo (Interferon Beta-1a) in Patients Having Acute Respiratory Distress Syndrome (ARDS)	Faron Pharmaceuticals Ltd	300
Biological therapy	NCT00789685	interferon-beta-1a	Safety, Tolerability and Preliminary Efficacy of FP-1201 in ALI and ARDS. Phase I/II	Faron Pharmaceuticals Ltd	37
Biological therapy	NCT01627613	Peptide mimicking the lectin-like domain of TNF	Study in Intensive Care Patients to Investigate the Clinical Effect of Repetitive Orally Inhaled Doses of AP301 on Alveolar Liquid Clearance in Acute Lung Injury	Apeptico Forschung und Entwicklung GmbH	40

For ARDS, no pharmacological treatment other than early paralyzation and prolonged steroids are routinely used at the bedside. This reality certainly demonstrates that we can identify targets and effective treatments in preclinical studies. However, we are unable to transfer the benefits to "real patients." In this context, we have to keep in mind that "clinical trials are not designed to demonstrate the effectiveness of a treatment in a random sample of the general population,"^(29)^ since drugs exert their effect on specific targets, and obviously the target has to be present in the cohort in which the drug is tried on.^(21)^ In other words, you can only lump patients who carry the same target. If not, you have to split them into subgroups of patients that carry the same target. Using this point of view, if only half of the patients with ARDS present DAD, and if most of the targets have been identified in animal models (in which the histology was considered as the gold standard), the high number of failing pharmacological treatments applied to ARDS cannot be a surprise.^(3,11,16,19,22)^

The term enrichment refers to the "prospective use of any patient´s characteristic to select a study population in which the detection of a drug effect (if one is present) is more likely than it would be in an unselected population."^(29)^ Here, the great interest lies in biomarkers, present in minimally invasive samples, such as serum, urine or bronchoalveolar lavage, to surrogate the diagnosis of DAD.

As previously mentioned, only early paralyzation^(30)^ and prolonged steroid therapy^(31)^ may be considered effective pharmacological treatment for severe ARDS. We hypothesize that this positive result may be related to the fact that they exert their effect over targets present in several entities that may mimic the ARDS.^(32,33)^ For that reason, it is possible to lump these entities in a clinical trial. Specifically, in the case of early paralyzation, the targets could be (a) the reduction in lung injury arising from ventilator desynchrony, (b) the attenuation of biotrauma and (c) limited expiratory muscle function, which reduces the respiratory system collapse and derecruitment.^(30)^ In addition, a recent experimental study suggests that neuromuscular blockers may inhibit the nicotinic pathway and induce an anti-inflammatory effect.^(34)^

**Figure 2:**
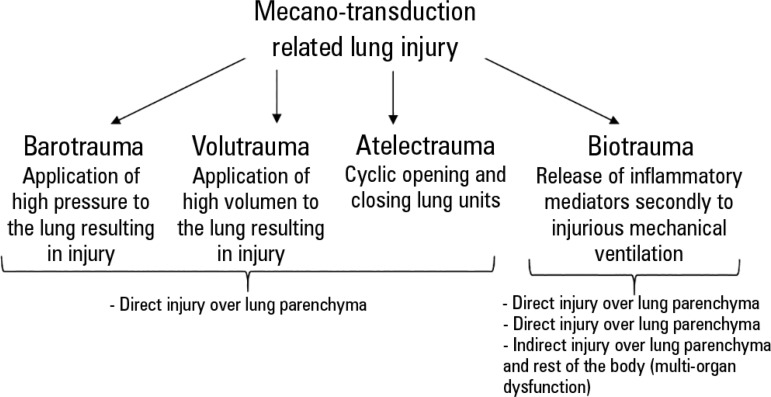
Mechano-transduction related lung injury.

For all of the above reasons, although not definitively, the most plausible mechanism to explain the beneficial effect of early paralyzation on ARDS outcome is the attenuation of mechano-transduction related to lung injury (Figure 2). This is non-specific to ARDS patients and may also benefit all subjects who require mechanical ventilation.^(35,36)^ On the other hand, the effectiveness of steroids in ARDS may be explained by at least three reasons: (a) the potent down-regulation of inflammatory and fibroproliferative pathways; (b) the benefit of steroids in pneumonia (this is the second most common histological pattern in patients with ARDS);^(37)^ and (c) other specific diseases that may mimic ARDS (e.g., acute eosinophilic pneumonia, diffuse alveolar hemorrhage from vasculitis, cryptogenic organizing pneumonia, acute hypersensitivity pneumonitis and pneumocystis jiroveci pneumonia).^(38)^

## CONCLUSION

Every intensivist should know that diffuse alveolar damage is present in only half of patients with acute respiratory distress syndrome. Based on recent discoveries, diagnosing diffuse alveolar damage is not merely an academic exercise because its effects on acute respiratory distress syndrome outcome have been demonstrated. At this moment, the only way to diagnose diffuse alveolar damage is to perform an open lung biopsy. However, recently, several efforts have been performed to identify a surrogate biomarker that would allow us to diagnose diffuse alveolar damage without the risk of open lung biopsy. Currently, N-terminal-peptide type III procollagen appears to be an accurate surrogate biomarker for the fibro-proliferative phase of acute respiratory distress syndrome. In coming years, it will be of paramount importance to validate N-terminal-peptide type III procollagen in a large cohort of patients with acute respiratory distress syndrome, as well as to seek out other molecular or imaging biomarkers able to surrogate the diagnosis of diffuse alveolar damage.
